# Dual Function of NAC072 in ABF3-Mediated ABA-Responsive Gene Regulation in *Arabidopsis*

**DOI:** 10.3389/fpls.2016.01075

**Published:** 2016-07-19

**Authors:** Xiaoyun Li, Xiaoling Li, Meijuan Li, Youcheng Yan, Xu Liu, Ling Li

**Affiliations:** ^1^Guangdong Provincial Key Lab of Biotechnology for Plant Development, College of Life Sciences, South China Normal UniversityGuangzhou, China; ^2^Key Laboratory of South China Agricultural Plant Molecular Analysis and Genetic Improvement, South China Botanical Garden, Chinese Academy of SciencesGuangzhou, China

**Keywords:** dual function, NAC072, ABF3, abscisic acid, RD29A, RD29B

## Abstract

The NAM, ATAF1/2, and CUC2 (NAC) domain proteins play various roles in plant growth and stress responses. *Arabidopsis* NAC transcription factor NAC072 has been reported as a transcriptional activator in Abscisic acid (ABA)-responsive gene expression. However, the exact function of NAC072 in ABA signaling is still elusive. In this study, we present evidence for the interrelation between NAC072 and ABA-responsive element binding factor 3 (ABF3) that act as a positive regulator of ABA-responsive gene expression in *Arabidopsis*. The transcript of *NAC072* is up-regulated by ABF3 in ABA response, and NAC072 protein interacts with ABF3. Enhanced ABA sensitivity occurs in *nac072* mutant plants that overexpressed *ABF3*. However, overexpression of *NAC072* weakened the ABA sensitivity in the *abf3* mutant plants, but instead of recovering the ABA sensitivity of *abf3*. NAC072 and ABF3 cooperate to regulate *RD29A* expression, but are antagonistic when regulating *RD29B* expression. Therefore, NAC072 displays a dual function in ABF3-mediated ABA-responsive gene regulation.

## Introduction

Abscisic acid (ABA) is an important signaling molecule that enables plants to tolerate unfavorable environmental stresses such as drought, salt, cold, heat, and oxidation (Hauser et al., 2011). Core components that consist of ABA signaling have been identified, including ABA receptors PYRABACTIN RESISTANCE (PYR/PYL; Park et al., 2009), PROTEIN PHOSPHATASE 2C (PP2C; Ma et al., 2009), SNF1-RELATED PROTEIN KINASE 2 (SnRK2; Nakashima et al., 2009), downstream transcription factors (TFs) such as ABA INSENSITIVE 3/4/5 (ABI3/4/5), ion channels, and NADPH oxidases (Mustilli et al., 2002; Schweighofer et al., 2004). In *Arabidopsis*, the ABA-responsive element binding factor (ABF/AREB) members (ABF1, AREB1/ABF2, AREB2/ABF4, and ABF3) can bind the ABA-responsive element (ABRE) and function in ABA-dependent signaling pathway (Yoshida et al., 2010). Principally, *ABF1* is in response to cold; overexpression of *ABF2* confers tolerance to drought, salt, heat, and oxidative stress; *ABF4* is mainly induced by drought, salt, and cold; and overexpression of *ABF3* confers tolerance to drought, salt, cold, heat, and oxidative stresses. ABF3 has largely overlapping functions with other ABF/AREB members and ABI5, and mainly acts in ABA-mediated response during seedling development (Finkelstein et al., 2005; Abdeen et al., 2010).

The NAC (NAM, ATAF1/2, and CUC2) TFs contain a plant-specific highly conserved N-terminal domain and play various roles in plant development, senescence and the response to environmental stress (Woo et al., 2004). The molecular function of NAC TFs in abiotic stress-responses has been examined in *Arabidopsis*. For example, ORESARA1 (ORE1/AtNAC2) positively regulates the salt stress response (Balazadeh et al., 2010); JUNGBRUNNEN1 (JUB1/NAC042) directly regulates dehydration responsive element binding protein 2A (DREB2A) and acts as a negative regulator of the salt stress response (Shahnejat-Bushehri et al., 2012); NAC016 promotes drought stress responses by repressing AREB1/ABF2 transcription (Sakuraba et al., 2015); and NAC096 directly interacts with ABF2 and ABF4 (but not with ABF3) to assist plant survival during dehydration and osmotic stress (Xu et al., 2013). These results suggest that NAC family members may serve as mediators through crosstalk with other stress regulators, such as the stress-responsive TFs.

NAC072/RD26, NAC019, and NAC055/AtNAC3 belong to the same clade of NAC domain genes and may have overlapping functions in the drought response (Hickman et al., 2013). Overexpression of these NAC TFs in plants enhances drought tolerance, indicating that they may act as positive regulators of the drought stress response (Fujita et al., 2004). Meanwhile, NAC019 and NAC055 are involved in jasmonic acid and/or ethylene signaling pathways, whereas NAC072 has been reported to be associated with the ABA-dependent stress response and to be strongly induced by ABA (Fujita et al., 2004; Tran et al., 2004). Double and triple mutants of these three homologous NAC genes have been obtained by crossing the single mutants with each other, and subsequent ABA response and downstream gene expression were analyzed with or without exogenous ABA treatments. Following exogenous ABA treatment, there was no significant difference in germination rate or root growth between the single mutants and the double mutants, although they were more ABA insensitive than the wild type (WT). The triple mutant of *nac019nac055nac072* was only slightly more sensitive to ABA or dehydration than the single mutants of *nac019, nac055*, and *nac072* or the double mutants of *nac019nac055, nac055nac072*, and *nac019nac072* (Lu et al., 2015). However, *NAC072* plays a dominant role in the development of green cotyledons, and *NAC055* coordinates with *NAC072* during this process to regulate the expression of ABA-responsive downstream genes (Lu et al., 2015). These findings indicate that NAC072, NAC019, and NAC055 may act individually in different stress responses, and that NAC072 is mainly responsible for ABA response.

Several TFs or downstream genes targeted by NAC family members have been identified. As previously mentioned, NAC016 directly represses the expression of *AREB1* (Sakuraba et al., 2015), and NAC096 binds ABF2/AREB1 and ABF4 (Xu et al., 2013), suggesting that the NAC family are closely involved with ABF/AREB TFs during senescence or stress responses. Promoter fragments of the *NAC072, NAC019*, and *NAC055* genes contain ABRE motifs, and was bound either ABF3 or ABF4 according to yeast one-hybrid assays (Hickman et al., 2013). This suggests that ABF/ABRE TFs may also regulate these NAC TFs. NAC072/RD26 has been reported to be involved in the ABA-mediated regulation of drought-responsive genes; however, the specific genetic effect of *NAC072* in the ABA signaling pathway is still unknown. Whether physical interactions occur between NAC072 and ABF/AREB TFs or other ABA-responsive signaling components, and subsequently involve in ABA response, remains unresolved.

Here, we show that the *NAC072* gene is upregulated by overexpression of ABF3. Loss of *NAC072* function results in ABA insensitivity, while loss of both *NAC072* and *ABF3* function further improve ABA insensitivity. Overexpression of *NAC072* enhances ABA sensitivity in WT plants, but does not recover the ABA sensitivity of the *abf3* mutant. This suggests that *NAC072* is positively involved in the ABA response and is dependent on ABF3. On the other hand, *nac072* mutants with overexpressed *ABF3* display greater ABA sensitivity than WT plants with overexpressed *ABF3*, indicating that *NAC072* is negatively involved in the ABF3-mediated ABA response. Furthermore, *NAC072* is regulated by ABF3, and NAC072 interacted with ABF3. In response to ABA, NAC072 cooperates with ABF3 to regulate *RD29A* expression, but acts antagonistically to ABF3 in the mediation of *RD29B* regulation. In summary, NAC072 displays a dual function in ABF3-mediated ABA signaling.

## Materials and Methods

### Plant Materials and ABA Application

The *Arabidopsis* seeds for *abf3* (SALK_17755), *nac072* (SALK_083756), *nac055* (SALK_014331), and *nac019* (SALK_096295) were obtained from the *Arabidopsis* Biological Resource Center (ABRC^1^). The seeds for *nac072abf3, nac055/72, nac019/72*, and *nac055/072/019* (*3P*) were obtained by hybridization. All seeds were surface sterilized in 70% ethanol containing 0.5% Tween-100. The supernatant was discarded and the seeds were suspended in ethyl alcohol and then air dried on filter paper. The seeds were sown on 0.5x MS medium with 0.8% agar containing 2% sucrose. After 2 days stratification at 4°C, the seeds were germinated and grown in a climate chamber under a daily cycle of 16 h light and 8 h dark at 20 ± 2°C. To examine the effects of ABA on germination and the development of green cotyledons, surface sterilized seeds were sown on 0.5x MS medium with 0.8% agar containing various concentrations of ABA (0, 0.5, 1, or 2 μM). The root growth experiment was conducted at 3 days after germination, for which the seedlings were transferred to new 0.5x MS media with 0.8% agar containing 10, 30, or 50 μM ABA.

### Plasmid Construction and *Arabidopsis* Transformation

The cDNAs encoding *ABF3* or *NAC072* were amplified and cloned into the modified *pCAMBIA1301* plasmids (*p35S: mCherry-6Myc* or *p35S:eGFP-3Flag*) to obtain *p35S:ABF3-mCherry-6Myc* or *p35S:NAC072-eGFP-3Flag*, respectively. The primers used to construct these plasmids are listed in Supplementary Table S1. The plasmids were transformed into *Arabidopsis* by the floral dip method using *Agrobacterium tumefaciens* strain GV3101 as previously reported (Li et al., 2013). Transgenic lines were screened using 0.5x MS medium containing 40 mg/L hygromycin (Sigma–Aldrich). The plasmids of *pRD29A:GUS* and *pRD29B:GUS* were provided by Hou XL Lab, south china botanical garden, CAS.

### Yeast Two-Hybrid Assay

To analyze the interactions between NAC072-F, NAC072-N, NAC072-C, and ABF3, the full NAC072-F and sectionalized sequences encoding NAC072-N (1–167aa) and NAC072-C (168–297aa) were amplified and cloned into pGBKT7 or pGADT7 (Clontech), respectively. The sequence encoding full-length ABF3 was amplified and cloned into pGADT7 or pGBKT7. The primers used to construct these plasmids are listed in Supplementary Table S1. All yeast transformants were grown on SD/-Trp/-Leu or SD/-Trp/-Leu/-His/-Ade (Clontech) medium for the interaction test.

### Expression Analysis

Two-week-old seedlings under control conditions or after various treatments were analyzed for gene expression using quantitative real time PCR analysis. Total RNA was extracted using a TRIzol Kit (TaKaRa), and a PrimeScript^TM^ RT reagent Kit (Perfect Real Time, TaKaRa) was used for reverse transcription. Real-time PCR was performed in an ABI 7500 system with the SYBR^®^ Premix DimerEraser^TM^ (Perfect Real Time, TaKaRa), each reaction consisted of 20 ng of cDNA, 0.1 μM primers and 10 μL 2x SYBR Premix ExTaq in a final volume of 20 μL. The reactive cycle used 95°C for 30 s, then 40 cycles at 95°C for 5 s and 60°C for 34 s. The gene expression quantity was calculated using the relative 2^-ΔΔCT^ method as previously reported (Li et al., 2013). The relative expression level was normalized to that of *ACT2* internal control. Primers used for gene expression analysis are listed in Supplementary Table S1. Assessment of NAC072 and ABF3 protein levels was carried out using western blot (WB) with Flag (F3165, Sigma) or c-Myc antibody (sc-40, Santa Cruz) as previously described (Sakuraba et al., 2015). The β-glucuronidase (GUS) activity of the *RD29A* and *RD29B* promoters was previously described (Woo et al., 2004). The presented data are the average of three technical replicates, and experiments were biologically repeated at least three times with similar results.

### Transient Transactivation Assay

To create the *pRD29A:GUS* or *pRD29B:GUS* reporter constructs, a ∼1200 bp region of the *RD29A* promoter or a ∼600 bp region of the *RD29B* promoter was cloned into pGreen vectors that was kindly provided by Hou XL Lab. The effector constructs (*p35S:ABF3-mCherry-6Myc* or *p35S:NAC072-eGFP-3Flag*) were generated by amplifying the corresponding cDNAs and then cloning into pGreen-35S. The *p35S:luciferase* (LUC) plasmid was used as an internal control to evaluate protoplast transfection efficiency. *Arabidopsis* mesophyll protoplasts were prepared and subsequently transfected as previously described (Sheen, 2001), after which GUS and LUC activities were analyzed and calculated. For each experiment, the 2 μg plasmids of *NAC072* or *ABF3* were used for tranfection in every group in same amount of protoplast. More than 80% transformation efficiency were confirmed by confocal microscopy using the difference fluorescent label protein (GFP for *NAC072*, mCherry for *ABF3*), respectively. The protein expression level of NAC072 and ABF3 was confirmed using WB with Flag or c-Myc antibody. The presented data are the average of three technical replicates, and experiments were biologically repeated at least three times with similar results.

### Bimolecular Fluorescence Complementation (BiFC) Assay

For analysis of interactions between ABF3 and NAC072, full-length cDNA of ABF3 and NAC072 were cloned into the *pGreen* binary vector containing C- or N-terminal fusions of EYFP, generated *p35S:ABF3-EYFP^C^* and *p35S:NAC072-EYFP^N^.* Then both of them cotransformed into *Arabidopsis* mesophyll protoplasts as previously described (Sheen, 2001). After darkness cultured for 24 h, the YFP fluorescence was determined using a confocal microscopy (Carl Zeiss LSM 710).

### Statistical Analysis

In order to evaluate the differences in data of physiological phenotype, gene expression and GUS reporter assay, student *T-*test was used to assess these data. Statistics quantitative data were expressed as mean ± SD. Means were compared using one-way analysis of variance or the student’s τ-test with SPSS13.0. Significance was assigned at *P* < 0.05.

### Accession Numbers

Sequence data from this article can be found in the *Arabidopsis* genome initiative database under the following accession numbers: *NAC072* (AT4G27410), *ABF3* (AT4G34000), *ACT2* (AT3G18780), *RD29A* (AT5G52310), *RD29B* (AT5G52300), *NAC055* (AT3G15500), and *NAC019* (AT1G52890).

## Results

### The Relationship between NAC072 and ABF3 in ABA Response and ABA-Inducible Gene Regulation

**FIGURE 1:**
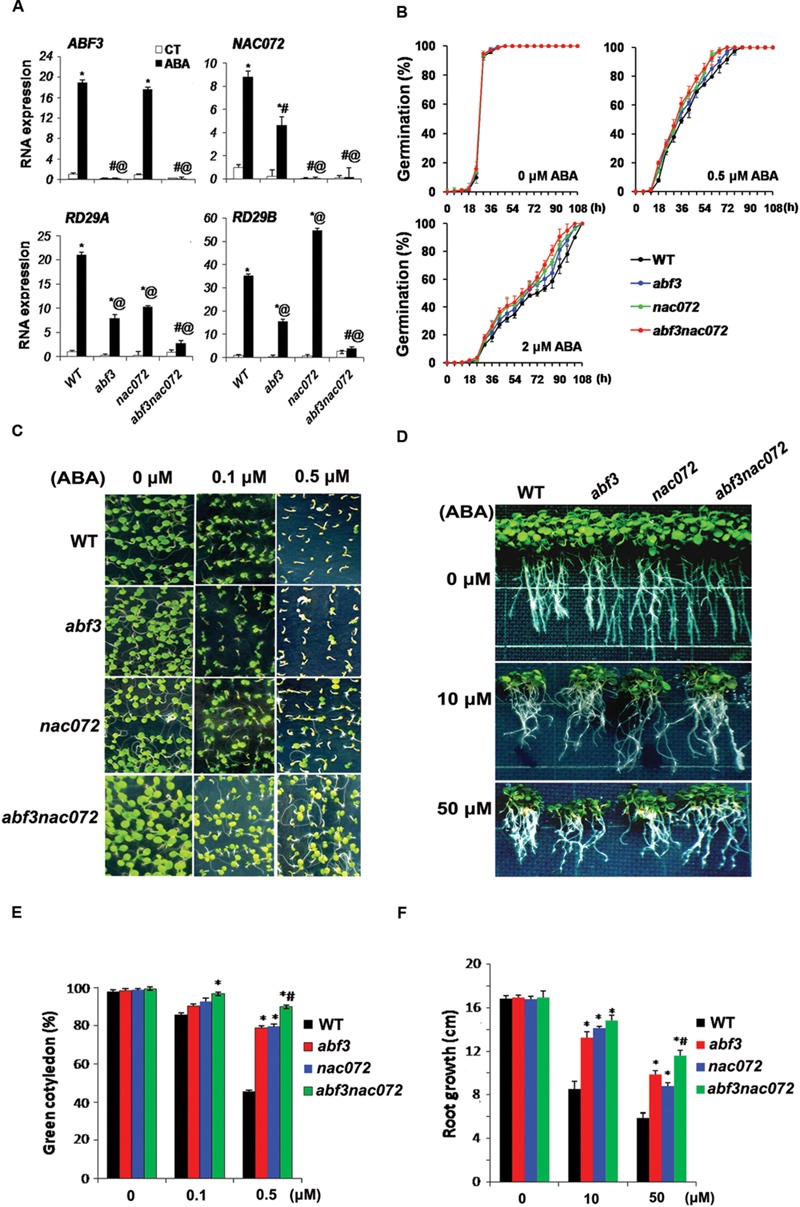
**The synergistic function of NAC072 in ABF3-mediated ABA response. (A)** Gene expression of *ABF3, NAC072, RD29A*, and *RD29B* in mutant (*abf3, nac072*, and *abf3nac072*) and wild type (WT) plants in response to ABA. The seedlings were grown on 0.5x MS agar plates for 12 days and then sprayed either with water (CT) or 100 μM ABA (ABA) and assayed after 2 h. Bars indicate standard deviation, *n* = 3. ‘^∗^’ indicates a significant difference between CT and ABA treatments (*P* < 0.05). ‘^#^’ indicates a significant difference between each mutant plants compared with WT plants under CT treatment (*P* < 0.05). ‘@’ indicates a significant difference between each mutants plants compared with WT plants under ABA treatment (*P* < 0.05). **(B)** Seed germination rate of mutant and WT in response to ABA. Germinations were recorded from 0 to 108 h after stratification on 0.5x MS agar plates containing 0, 0.5, or 2 μM ABA. **(C)** Photographs of seedlings grown for 7 days after stratification on agar plates containing 0, 0.1, or 0.5 μM ABA. **(D)** Photographs of seedlings grown for 20 days after transferral to control agar plates (0 μM ABA) or plates containing 10 or 50 μM ABA. **(E)** The statistical analysis of green cotyledons in **(C)**. Bars indicate standard deviation, *n* = 30. **(F)** The statistical analysis of axial root growth in **(D)**. Bars indicate standard deviation, *n* = 10. ‘^∗^’ indicates a significant difference between *abf3* or *nac072* plants compared with WT plants under the same ABA treatment (*P* < 0.05). ‘^#^’ indicates a significant difference between *abf3nac072* plants compared with *abf3* or *nac072* plants (*P* < 0.05).

Previous researches (Fujita et al., 2004; Tran et al., 2004) and our own observations confirm that overexpression of *NAC072* improves ABA sensitivity in *Arabidopsis*, whereas loss of *NAC072* function in plants reduces ABA sensitivity (Supplementary Figure S1). This indicates that NAC072 is a positive regulator for ABA signaling. To examine the relationship between NAC072 and ABF3, the expression of *NAC072* was first analyzed in the loss-of-function mutant of *ABF3* (*abf3*). The *NAC072* expression was dramatically reduced in *abf3* after ABA treatment, whereas the transcript level of *ABF3* was not affected in the *NAC072* deficient mutant *nac072* (**Figure 1A**). It suggests that the expression of *NAC072* is mediated by ABF3 under ABA treatment. Meanwhile, *RD29A* and *RD29B* are rapidly induced by drought stress or ABA, and are thus considered to be stress-response marker genes (Msanne et al., 2011). Both *RD29A* and *RD29B* were highly upregulated by ABA treatment, while their expressions were dramatically reduced in *abf3nac072* mutants, although different expression patterns were observed in the single mutants. *RD29A* was repressed in *abf3* and *nac072* after ABA treatment; although *RD29B* was attenuated in *abf3* mutants, it increased notably in *nac072* after ABA treatment (**Figure 1A**). These suggest that NAC072 may cooperate with ABF3 in the regulation of *RD29A* gene expression, while acting antagonistically with ABF3 in the regulation of *RD29B* gene expression. Thus, NAC072 seems to play a dual role in ABA-inducible gene expression in combination with ABF3.

To examine this implication, ABA sensitivity was further assessed in *abf3, nac072*, and *abf3nac072* mutants by the physiological experiments on germination, the development of green cotyledons and axial root growth. During the germination process, no significant difference was observed between WT, *abf3, nac072*, and *abf3nac072* under 0, 0.5, or 2 μM of ABA treatments (**Figure 1B**). However, remarkably, these mutants showed ABA insensitivity during the development of green cotyledons and axial root growth compared with the WT plants, especially the double mutant *abf3nac072* displayed the greatest ABA insensitivity (**Figures 1C–F**). All of the above results indicate that NAC072 partly cooperates with ABF3 in the ABA response during the development of green cotyledons and axial root growth, but not during the germination process. However, it seems contradictory that there is cooperation between NAC072 and ABF3 with respect to physiological function, yet there is antagonism between these TFs in the mediation of ABA-inducible expression of genes such as *RD29B*. Therefore, NAC072 may perform a dual function in ABF3-mediated gene regulation of the ABA response.

### NAC072 Cooperates with and Partly Depends on ABF3 Involvement in ABA Signaling

**FIGURE 2:**
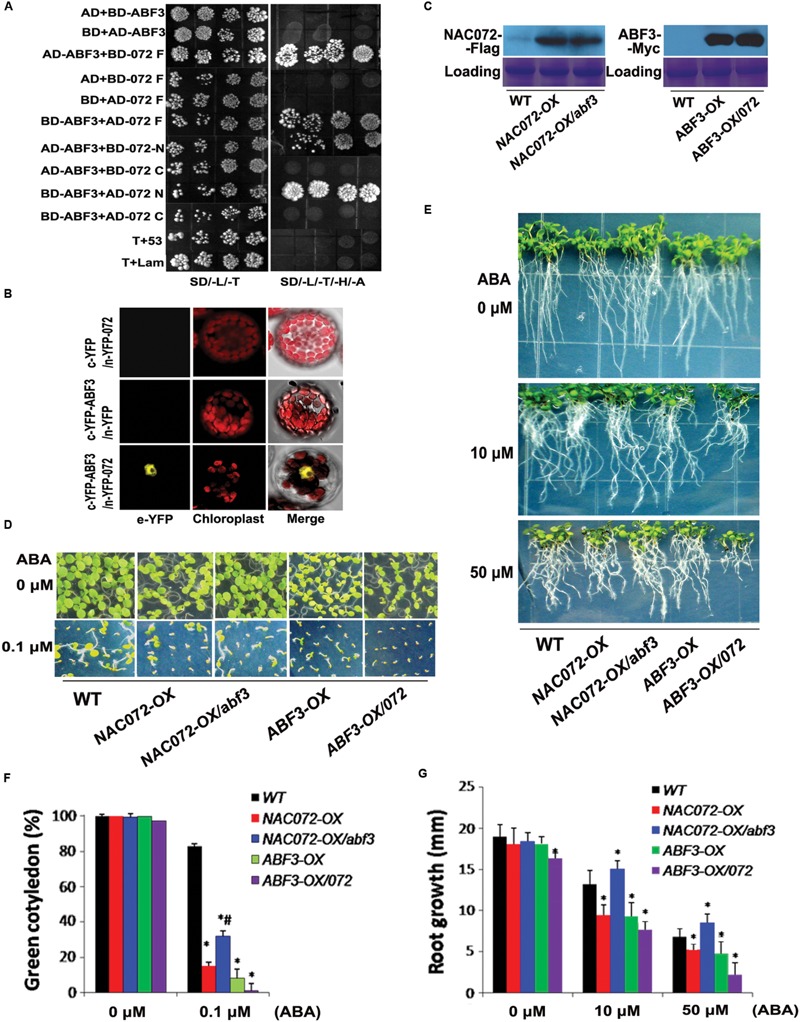
**The dual function of NAC072 in ABF3-mediated ABA response. (A)** Interactions between ABF3 and both full length and sections (C- and N-terminal regions) of NAC072 were confirmed by yeast two-hybrid assay. The 072 F indicates NAC072 full length, 072 N or C indicates NAC072 N-terminal regions or C-terminal regions, respectively. T+53 and T+Lam were used as the negative controls. **(B)** Interactions of ABF3 and NAC072 were observed by BiFC assay. **(C)** NAC072 or ABF3 protein accumulation in the transgenic plants detected by western blot. **(D)** Photographs of seedlings recorded at 7 days after stratification on agar plates containing 0, or 1 μM ABA. **(E)** Photographs of seedlings recorded at 20 days after transferral to control agar plates (0 μM ABA) or plates containing 10 or 50 μM ABA. *NAC072-OX* or *ABF3-OX* indicates *NAC072* or *ABF3* overexpressed in WT under the control of the cauliflower mosaic virus (CaMV) 35S promoter, respectively. *NAC072-OX*/*abf3* or *ABF3-OX*/*072* indicates *NAC072* or *ABF3* overexpressed in *abf3* or *nac072*, respectively. **(F)** The statistical analysis of green cotyledons in **(D)**. Bars indicate standard deviation, *n* = 30. **(G)** The statistical analysis of axial root growth in **(E)**. Bars indicate standard deviation, *n* = 10. ‘^∗^’ indicates a significant difference between *ABF3* or *NAC072* transgenic plants compared with WT plants under the same ABA treatment (*P* < 0.05). ‘^#^’ indicates a significant difference between *NAC072-OX*/*abf3* compared with *NAC072-OX* plants or *ABF3-OX*/*072* compared with *ABF3-OX* plants (*P* < 0.05).

Several NAC proteins interact with the ABF/AREB TFs involved in stress response signaling, including NAC016 and NAC096 (Xu et al., 2013; Sakuraba et al., 2015). Here, the interaction of NAC072 and ABF3 was demonstrated using the yeast two-hybrid system and BiFC assay. The results displayed that the full (NAC072-F) and N-terminal region of NAC072 (072-N) showed a stronger interaction with ABF3 in yeast (**Figure 2A**), and NAC072 and ABF3 also interact with each other in the nucleus (**Figure 2B**, Supplementary Figure S6). These data support the physical interactions between NAC072 protein and ABF3 TFs.

*NAC072* was overexpressed under the control of the cauliflower mosaic virus 35S (35S) promoter in WT plants. *NAC072-OX/abf3* plants were obtained by crossing *NAC072-OX* transgenetic line with the *abf3* mutant. For comparison, *ABF3-OX/072* plants were produced by crossing *ABF3-OX* with the *nac072* mutant. All homozygotes were determined by hygromycin resistance screening and tri-primer PCR after F2 generation segregation (data not shown). Accumulations of NAC072 or ABF3 in these plants were shown by western blot using antibodies against a Flag or c-Myc protein tag (**Figure 2C**). Thus, ABA sensitivities of these plants were examined during the development of green cotyledon and axial root growth. Overexpression of *NAC072* or *ABF3* (in the *NAC072-OX* or *ABF3-OX* plants) resulted in slight growth inhibition and enhanced ABA sensitivity (**Figures 2C,D**; Supplementary Figures S1, S2 and S5). However, overexpression of *NAC072* in *abf3* (*NAC072-OX/abf3*) resulted in less ABA sensitivity than in *NAC072-OX* or WT plants, and *NAC072-OX/abf3* had less ABA sensitivity than *abf3* (**Figures 2C,D**; Supplementary Figures S3, S5, and S7). This suggests that NAC072 is partly dependent on ABF3 for the positive regulation in ABA response, and NAC072 had a negative role on ABA response. Furthermore, the *ABF3-OX/nac072* plants (where ABF3 was overexpressed in the *nac072*) showed further improved ABA sensitivity compared with *ABF3-OX* or WT plants (**Figures 2C–G**; Supplementary Figures S4, S5 and S7). These results indicate that NAC072 antagonizes ABF3 during the development of green cotyledon or axial root growth under ABA treatment. In addition, overexpression of ABF3 in *nac019nac072, nac055nac072*, or *nac055nac072nac019* resulted in the same degree of ABA sensitivity as the *ABF3-OX/nac072* plants, but they displayed higher ABA sensitivity than *ABF3-OX* plants (Supplementary Figure S5). These further suggest that NAC072 has a dual role in the ABA response.

### NAC072 Antagonizes ABF3-Mediated ABA-Responsive Genes Regulation

**FIGURE 3:**
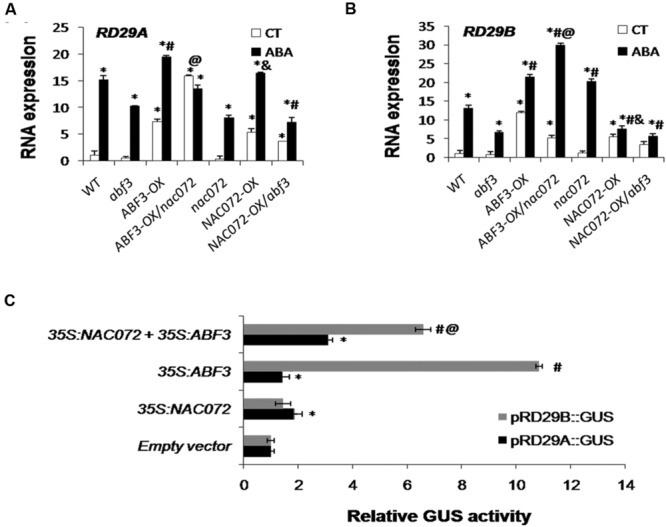
**NAC072 cooperates with or antagonizes ABF3-mediated ABA-responsive gene regulation. (A)** Expression of *RD29A* in each mutant, transgenic line and WT plants under control treatment (CT) or 100 μM ABA treatment (ABA) for 2 h. ‘^∗^’ or ‘^#^’ indicates a significant difference between *ABF3* or *NAC072* transgenic plants compared with WT or *abf3* and *nac072* plants under CT and ABA treatments, respectively (*P* < 0.05). ‘@’ indicates a significant difference between *ABF3-OX* and *ABF3-OX/nac072* plants (*P* < 0.05). ‘&’ indicates a significant difference between *NAC072-OX* and *NAC072-OX/abf3* plants (*P* < 0.05). **(B)** *RD29B* in each mutant, transgenic and WT plants under CT or ABA treatment for 2 h. ‘&’ indicates a significant difference between *NAC072-OX* and *nac072* mutants. **(C)** Transactivation of the *RD29A* or *RD29B* promoter-GUS fusion gene by ABF3 and/or NAC072 in *Arabidopsis* protoplasts. The reporter gene was transfected with each effecter plasmid (*35S:ABF3* and/or *35S:NAC072*) or the empty vector as a control groups. Co-transfection of the 35S driving *luciferase* (*LUC*) plasmid was used in each experiment to normalize transfection efficiency. Protoplasts were prepared from rosette leaves of 4-weeks-old WT. Bars indicate standard deviation, *n* = 3. ‘^∗^’ or ‘^#^’ indicates a significant difference between *ABF3* or *NAC072* groups compared with control groups (*P* < 0.05). ‘@’ indicates a significant difference between *ABF3* and *NAC072* compared with *ABF3* alone (*P* < 0.05).

To understand the crosstalk between NAC072 and ABF3 in ABA signaling, the regulations of NAC072 and ABF3 on *RD29A* and *RD29B* expression were further analyzed in different genotype combinations. Overexpression of *NAC072* or *ABF3* in WT plants (*NAC072-OX* or *ABF3-OX*) increased the transcription level of both *RD29A* and *RD29B*. In *NAC072-OX*/*abf3* plants, *RD29A* and *RD29B* expressions were enhanced under control conditions, but little change was observed after ABA treatment (**Figure 3A**). This suggests that, under ABA treatment, without ABF3, NAC072 cannot enhance the expression of *RD29A* or *RD29B.* That is *NAC072* regulated the expression of *RD29A* and *RD29B* after ABA treatment need the ABF3 mediation. On the other hand, the loss of *NAC072* function further contributed to ABF3-enhanced *RD29B* expression in *ABF3-OX/nac072* plants, but there was no obvious change in *RD29A* expression (**Figures 3A,B**). These findings add further confirmation that NAC072 is antagonistic to ABF3-mediated *RD29B* gene expression.

The expression regulation of *RD29A* and *RD29B* by ABF3 and/or NAC072 was further analyzed using GUS reporter systems. The 1200 bp region of the *RD29A* promoter and 600 bp region of the *RD29B* promoter were each fused with the GUS reporter gene. Either *NAC072* or *ABF3*, driven by the 35S promoter, was co-expressed with each GUS construct in protoplasts. The result showed the *RD29A* promoter fusion with the GUS gene was more strongly induced by *p35S::ABF3* together with *p35S::NAC072* than by *p35S::ABF3* or *p35S::NAC072* alone (**Figure 3C**), indicating that NAC072 cooperates with ABF3 to mediate the expression of the *RD29A* gene. A different regulation pattern was observed for *RD29B* expression. The single *p35S::ABF3* expression resulted in a stronger transactivation of *RD29B* compared with *p35S::NAC072* alone, or with *p35S::ABF3* and *p35S::NAC072* combined (**Figure 3C**). These results further indicate that NAC072 acts antagonistically with ABF3 in regulating the expression of *RD29B*. Taken together, NAC072 may function as a cofactor, which not only cooperates with but also antagonizes ABF3-mediated regulation of the ABA response.

## Discussion

In our previous work, we obtained double and triple mutants of three *Arabidopsis* NAC homologous genes (*NAC019, NAC055*, and *NAC072*). ABA sensitivity analysis indicated that NAC055 plays an important role during germination process, while NAC072 partly inhibited the expression of *RD29B* and *RAB18*, both of which are widely described as ABA signaling marker genes as their expressions are always induced by dehydration or ABA (Tran et al., 2004; Lu et al., 2015). However, NAC072 is reportedly involved in the ABA-dependent stress-signaling pathway and functions as a transcriptional activator in ABA-inducible gene expression (Fujita et al., 2004). These contradictory results prompted us to further explore the role of NAC072 in ABA signaling.

ABF3 is the key downstream regulator of ABA signaling and is important for ABA-mediated main root growth. Reprogramming the drought response by changing the timing or strength of expression of some drought-responsive genes was observed in plants with overexpressed *ABF3* (Finkelstein et al., 2005). ABA sensitivity in the *abf3* mutant was lower than that in the *abf2, abf4* mutants or WT during the development of cotyledon or main root growth (data not shown). Therefore, the ABF3-mediated ABA signaling pathway was chosen to illuminate the role of NAC072 in the ABA response. Subsequently, we uncovered four key points as follows: (1) the transcription level of *NAC072* is partly regulated by ABF3; (2) NAC072 interacts and cooperates with ABF3 to regulate gene expression (including that of *RD29A*) in the ABA response; (3) NAC072 partly depends on ABF3 for its role in the ABA response during the development of cotyledon and main root growth; (4) NAC072 antagonizes the action of ABF3 to some extent in the regulation of *RD29B* expression.

Transcriptome data from plants overexpressing ABF3 show that the expression of *NAC072/RD26* as well as *NAC019* and *NAC055/AtNAC3* is independent of ABF3 (Abdeen et al., 2010). In accordance with these results, we detected NAC072 expression in both *ABF3*-overexpressing plants and *abf3* mutants. However, either under control conditions or after ABA treatment, overexpression of *ABF3* increased the expression of *NAC072* (data not shown). Loss of ABF3 function reduced *NAC072* transcript levels, but the expression of *NAC072* could still be detected after ABA treatment (**Figure 1A**). Hence, the expression of *NAC072* seems partly dependent on ABF3.

ABF3, AREB2, and AREB1 can form homo- or heterodimers and have redundant function (Yoshida et al., 2010). Some NAC proteins have been observed to interact with the AREB family. Typically, ANAC096 directly interacts with ABF2/AREB1 and AREB2/ABF4, and NAC016 interacts with ABF2/AREB1 (Xu et al., 2013; Sakuraba et al., 2015). The relationship between NACs and ABF3 is still unclear, but our yeast two-hybrid results show that the C- or N-terminal regions of NAC072 interact with ABF3 (**Figure 2A**). It seems likely, then, that the contradictory roles of NAC072 in ABA signaling might be resolved by an understanding of its relationship with ABF3. We assumed that NAC072 functions as a cofactor with ABF3 in the ABA signaling pathway. Accordingly, ABA sensitivity and ABA-inducible genes were analyzed in *abf3, nac072*, and *abf3nac072* mutants (**Figure 1**). The results indicate that NAC072 cooperates with ABF3 in mediation of *RD29A* expression, but antagonizes *RD29B* expression. It is well known that both *RD29A* and *RD29B* rapidly respond to various stresses and ABA treatment (Narusaka et al., 2003). In the present study, *RD29B* was directly and positively regulated by ABF3, while NAC072 may act as a repressor during this process. To further confirm this, *NAC072* was overexpressed in WT and *abf3* mutant plants. The expression of *RD29B* was enhanced in *NAC072-OX/WT* and *NAC072-OX/abf3* plants under control conditions, but no change in expression occurred after ABA treatment. In contrast, overexpressed *ABF3* rapidly increased *RD29B* transcription levels in control as well as ABA-treated plants. The combined loss of NAC072 function and ABF3 overexpression contributed to enhanced *RD29B* expression. Moreover, *NAC072-OX/abf3* plants were more ABA insensitive than WT or *NAC072-OX/WT* plants, whereas *ABF3-OX/nac072* was more ABA sensitive than *WT* or *ABF3-OX/WT* plants. In addition, NAC072 collaborates with ABF3 to regulate *RD29A* expression. Thus, we hypothesize that NAC072 and ABF3 may act as a “push-pull” device. Under certain conditions, NAC072 cooperates with ABF3-mediated gene expression, thus providing a “push” stimulus, while under other conditions it acts antagonistically to ABF3, giving a “pull” effect. How the device works would depend on specific conditions, a detailed understanding of which requires further investigation.

## Author Contributions

XyL, XuL, and LL designed the research. XL, XlL, and YY performed research. XyL and XlL analyzed data. XyL and XuL wrote the article. The protein interaction experiments mainly performed for the revisions draft by XyL and ML.

## Conflict of Interest Statement

The authors declare that the research was conducted in the absence of any commercial or financial relationships that could be construed as a potential conflict of interest.

## Footnotes

1 http://www.arabidopsis.org
