# Supplementary material for: Dual Function of NAC072 in ABF3-Mediated ABA-Responsive Gene Regulation in Arabidopsis
Source: Front Plant Sci. 2016 Jul 19;7:1075. doi: 10.3389/fpls.2016.01075 (PMC4949229; doi:10.3389/fpls.2016.01075)
Supplement: Supplementary file 1 [file Presentation_1.PDF]

Supplemental Data

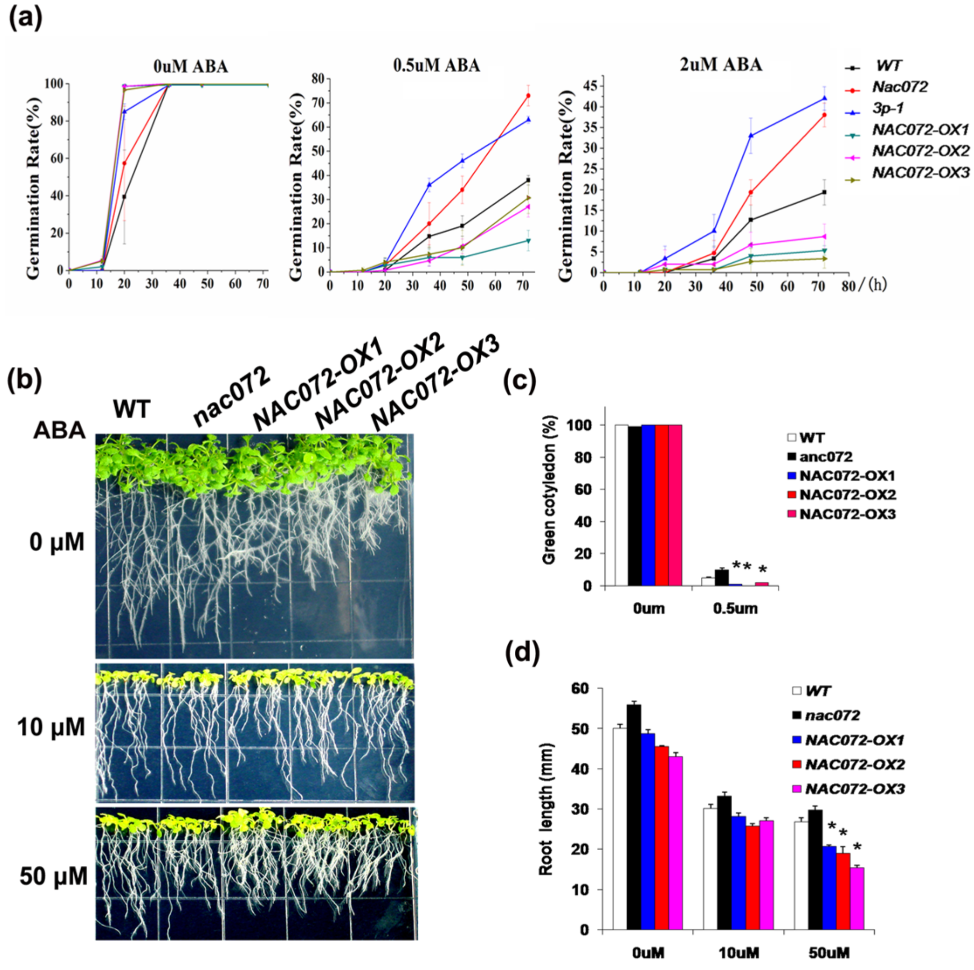

Figure S1 Overexpression of *NAC072* enhances ABA sensitivity in WT plants. Transgenic plants (*NAC072-OX1*, *NAC072-OX2* and *NAC072-OX2*) are more sensitive to ABA than WT or *nac* mutants during germination, green cotyledon development, or root growth. **(a)** Seed germination rates analyzed on 0.5× MS agar plates containing 0, 0.5, or 2 µM ABA. **(b)** Photographs of seedlings grown for 20 d after transferral to control agar plates (0 µM ABA) or plates containing 10 or 50 µM ABA. **(c)** Green cotyledons of WT, the *nac072* mutant and three transgenic lines (*NAC072-OX1*, *NAC072-OX2* and *NAC072-OX3*) counted 7 d after stratification. **(d)** Statistics of the relative primary root length of seedlings treated with 10, 30 or 50 µM ABA for 20 d after transferral to new plates. *NAC072-OX*: *NAC072* overexpressed in WT under the control of the 35S promoter. *3P-1* represents the *nac019nac055nac072* triple mutant. Bars indicate standard deviation, $n = 30$. '*' indicates a significant difference between *NAC072* transgenic plants compared with WT plants under ABA treatment ($P<0.05$).

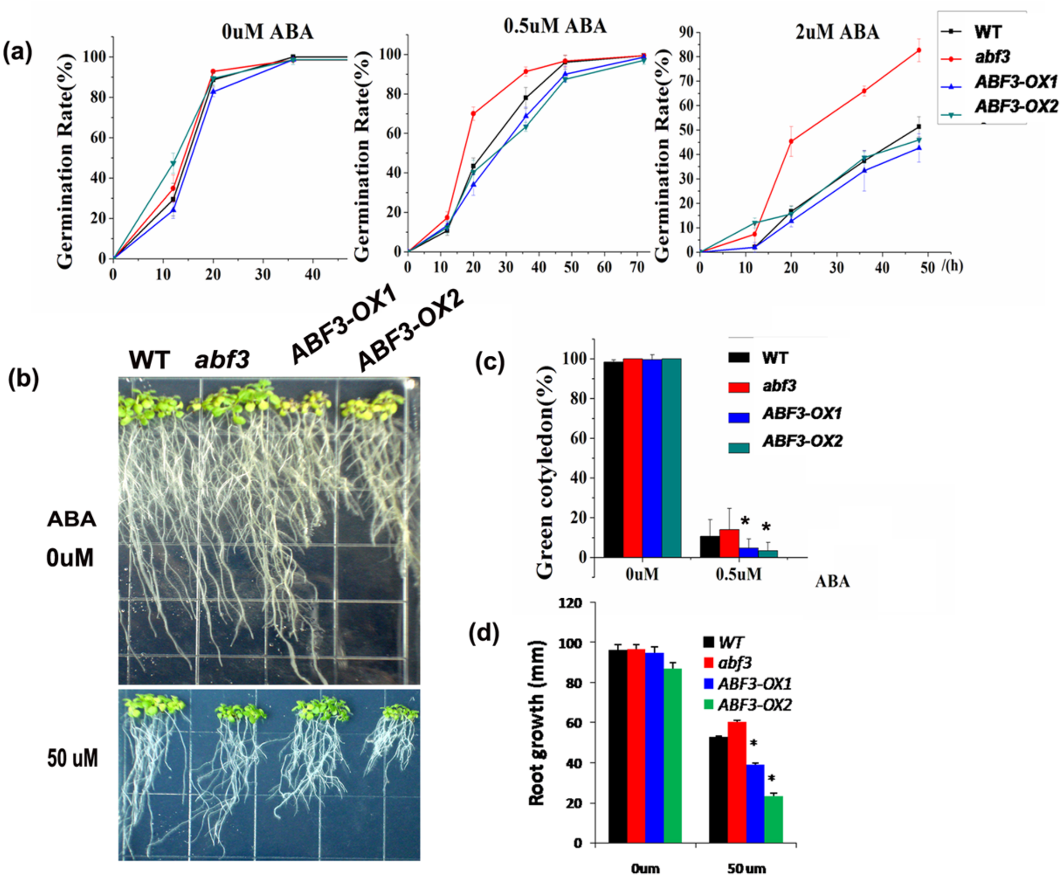

Figure S2 Overexpression of *ABF3* enhances ABA sensitivity in WT plants. The *ABF3-OX* plants are more sensitive to ABA than WT or *abf3* plants during germination, green cotyledon development or root growth. **(a)** Seed germination rates analyzed on 0.5 × MS agar plates containing 0, 0.5, or 2 μM ABA. **(b)** Photographs of seedlings grown for 20 d after transfer to agar plates (0 μM ABA) or plates containing 50 μM ABA. **(c)** Green cotyledons of WT, *abf3* and two transgenic lines (*ABF3-OX1* and *ABF3-OX2*) counted 7 d after stratification. **(d)** Quantification of relative primary root length of seedlings treated with 0 and 50 μM ABA on 20 d after transferral to new plates. *ABF3-OX*: *ABF3* overexpressed in WT under the control of the 35S promoter. Bars indicate standard deviation, $n = 30$. '*' indicates a significant difference between *ABF3* transgenic plants compared with WT or *abf3* plants under ABA treatment ($P<0.05$).

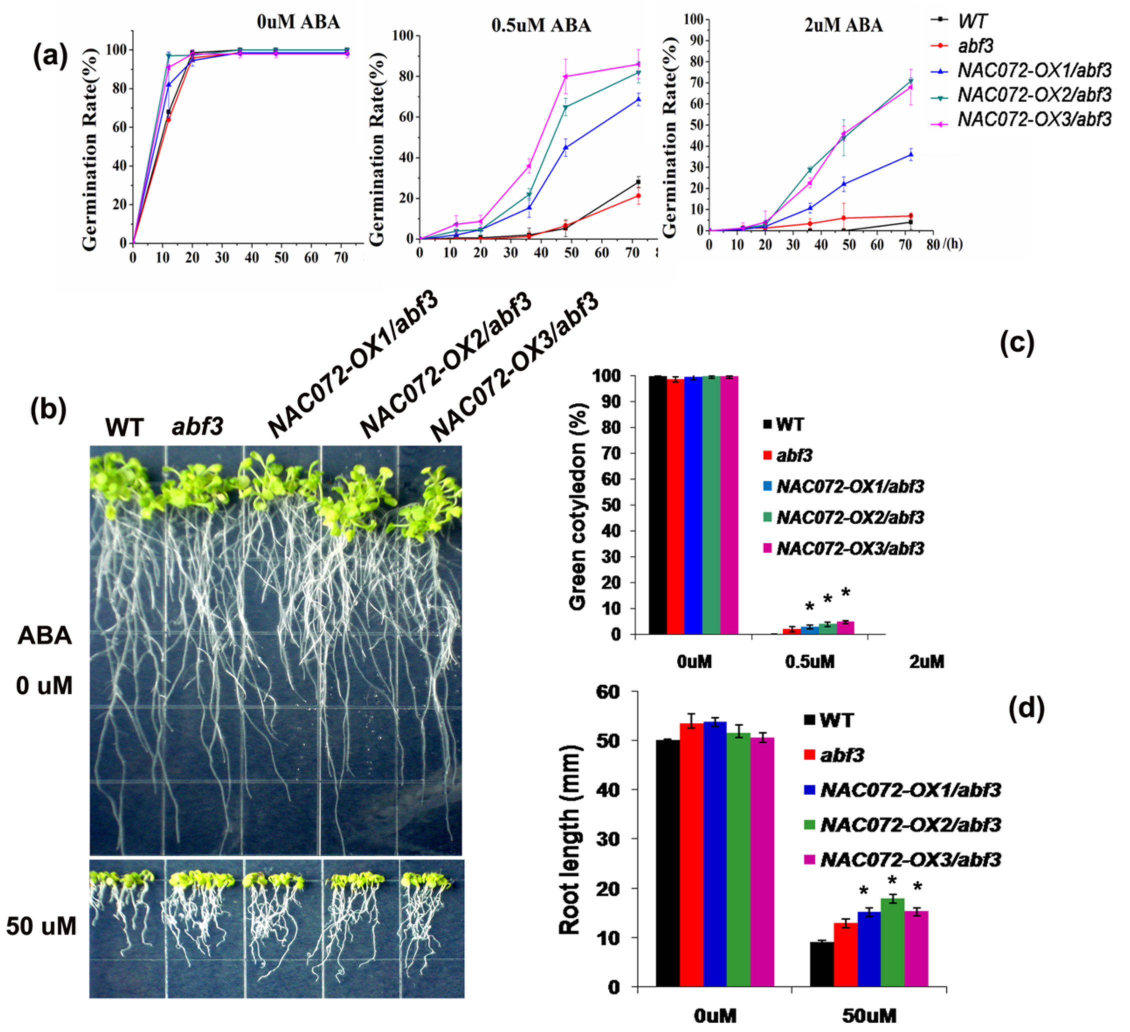

Figure S3 Overexpression of *NAC072* does not recover the ABA sensitivity of the *abf3* mutant. The *NAC072-OX/abf3* plants display less ABA sensitivity than WT or *abf3* plants during germination, green cotyledon development or root growth. **(a)** Seed germination rates of WT, *abf3*, and three *NAC072-OX/abf3* lines were analyzed on 0.5 × MS agar plates containing 0, 0.5, or 2 μM ABA. **(b, d)** Photographs and quantification of the relative primary root length of seedlings treated with 50 μM ABA 20 d after transferral to new plates. *NAC072-OX*: *NAC072* overexpressed in WT plants under the control of the 35S promoter. *NAC072-OX/abf3*: *NAC072* overexpressed in the *abf3* mutant after crossing the independent *NAC072-OX* transgenic line with the *abf3* mutant. **(c)** Green cotyledons of WT, *abf3* and three transgenic lines of overexpressed *NAC072* in *abf3* (*NAC072-OX1/abf3, NAC072-OX2/abf3* and *NAC072-OX3/abf3*) were calculated 7 d after stratification. Bars indicate standard deviation, n = 30. '*' indicates a significant difference between *NAC072-OX/abf3* transgenic plants compared with WT plants ($P<0.05$).

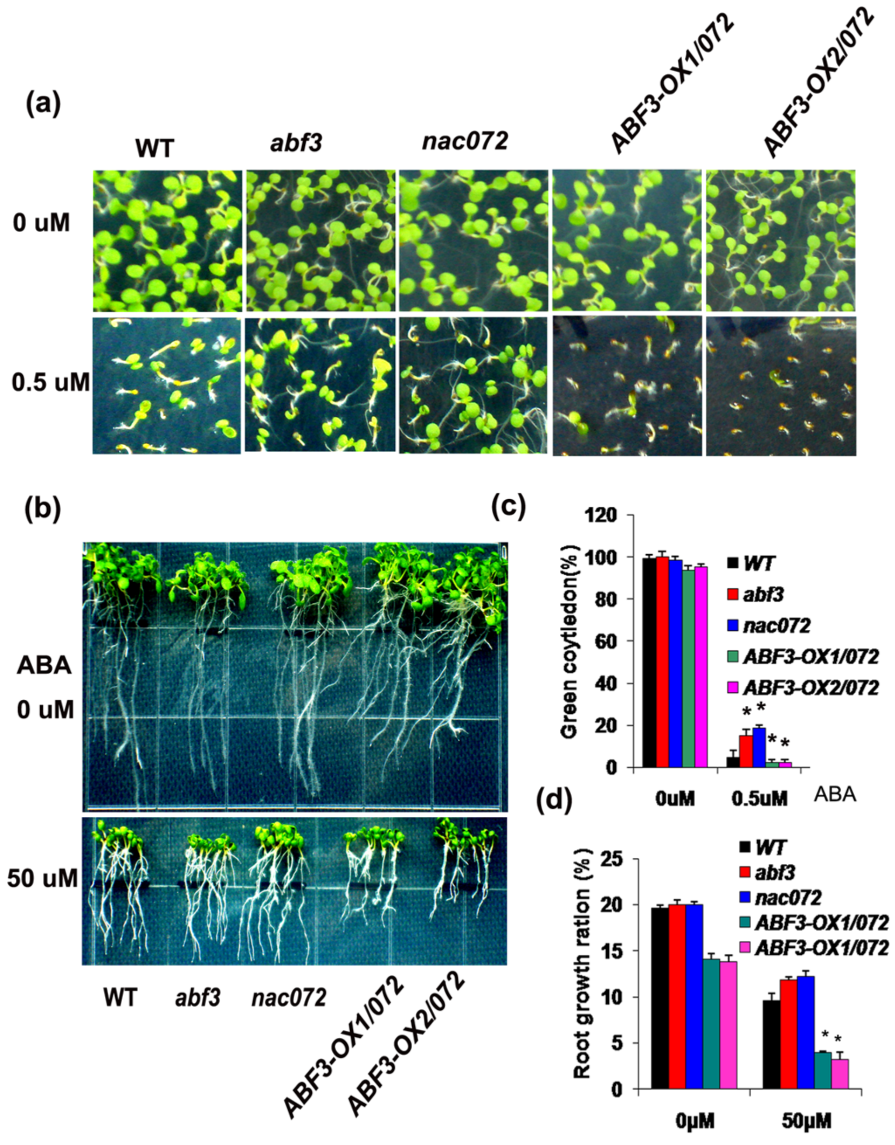

Figure S4 Enhanced ABA sensitivity in *nac072* mutants with overexpressed *ABF3*. **(a, c)** Green cotyledons of WT, *abf3, nac072* and two transgenic lines of overexpressed *ABF3* in *nac072* (*ABF3-OX1/072* and *ABF3-OX2/072*) were photographed and counted 7 d after stratification. *ABF3-OX1/072* and *ABF3-OX2/072* plants displayed more sensitivity to ABA than WT, *abf3*, or *nac072* plants. **(b, d)** Photographs and statistics of relative primary root length of seedlings treated with 0 and 50 μM ABA on 20 d after transferral to new plates. *ABF3-OX*: *ABF3* overexpressed in WT plants under the control of the 35S promoter. *ABF3-OX/072*: *ABF3* overexpressed in the *nac072* mutant after crossing *ABF3-OX* transgenic lines with the *nac072* mutant. Bars indicate standard deviation, n = 30. '*' indicates a significant difference between *ABF3* or *NAC072* transgenic plants or mutants compared with WT plants under control conditions or after ABA treatment, respectively ($P<0.05$).

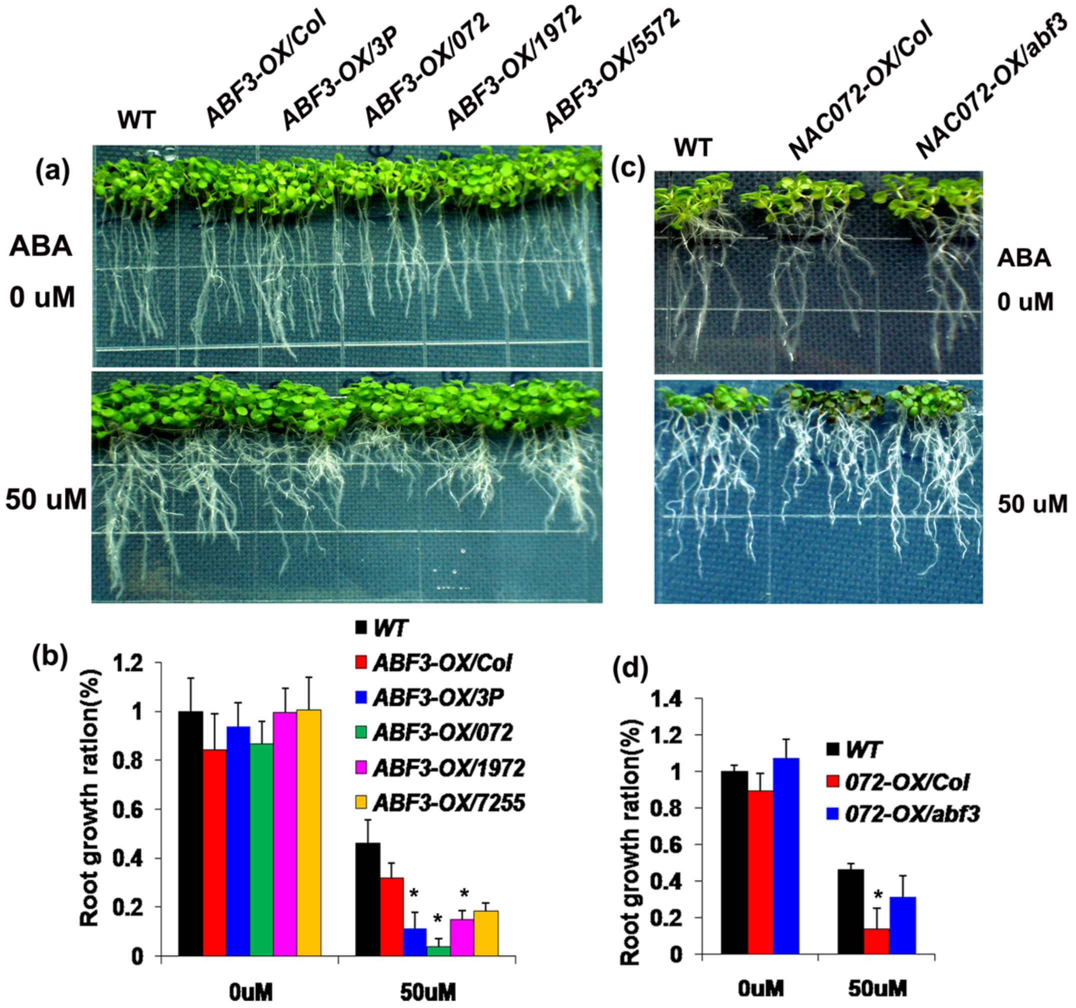

Figure S5 ABA sensitivity of various transgenic lines. Overexpression of *ABF3* in *nac072* (*072*), *nac072nac055* (*5572*), *nac019nac072* (*1972*), or *nac072nac055nac019* (*3P*) mutants enhances sensitivity to ABA during root growth, and *ABF3-OX/nac072* displays the most sensitivity to ABA; the ABA sensitivity is also increased by overexpressing *NAC072* in WT, but not in the *abf3* mutant. **(a, c)** Photographs of seedlings grown 20 d after transfer to agar plates (0 µM ABA) or plates containing 50 µM ABA. **(b, d)** Statistics of relative primary root length of seedlings treated with 0 and 50 µM ABA for 20 d after transferral. *ABF3-OX*: *ABF3* overexpressed in WT (Col) plants under the control of the 35S promoter. *ABF3-OX/3P*, *ABF3-OX/072*, *ABF3-OX/1972*, or *ABF3-OX/5572*: *ABF3* overexpressed in *nac072nac055nac019*, *nac072*, *nac019nac072*, or *nac055nac072*, after crossing the independent *ABF3-OX* transgenic line with these mutants, respectively. Bars indicate standard deviation, n = 30. '*' indicates a significant difference between *ABF3* or *NAC072* transgenic plants compared with WT plants under control conditions or after ABA treatment ($P<0.05$).

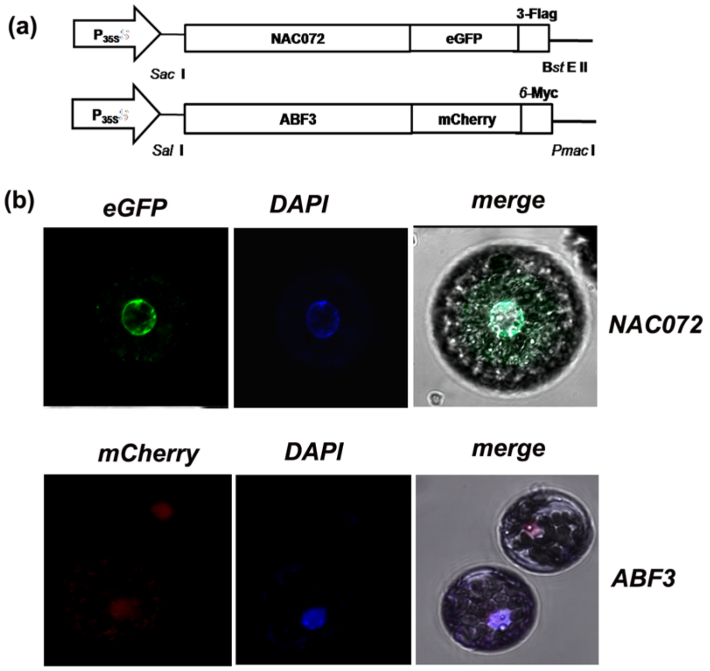

Figure S6 Subcellular localization of NAC072 and ABF3. NAC072-eGFP or ABF3-mCherry fusion proteins were transiently transformed into *Arabidopsis* protoplasts and observed using confocal microscopy. **(a)** The chemic constructs of *NAC072*-eGFP and *ABF3*-mCherry. **(b)** NAC072 and ABF3 are located in the nucleus (overlapped with DAPI stain), which is consistent with previous research. This validates use of the *NAC072* and *ABF3* constructs in subsequent experiments.

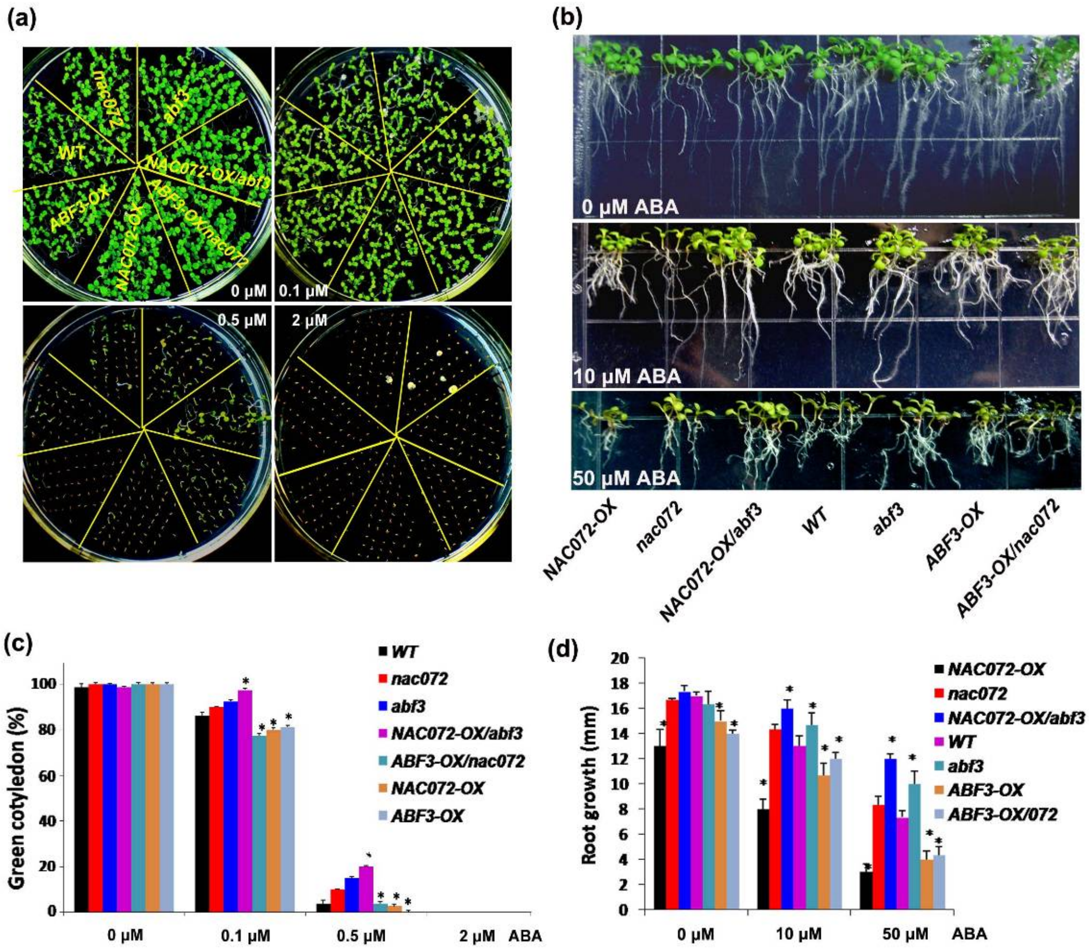

Figure S7 Repetition test of ABA sensitivity in various transgenic lines and mutants on single plate with different concentrations of ABA. (a) Photographs of seedlings grown for 7 d after stratification on agar plates containing 0, 0.1, 0.5 and 2 μM ABA. **(b)**. Photographs of seedlings recorded at 10 d after stratification on agar plates containing 0, 10 and 50 μM ABA. **(c)** The statistical analysis of green cotyledons in (a). Bars indicate standard deviation, *n* = 50. **(d)** The statistical analysis of axial root growth in (b). Bars indicate standard deviation, *n* = 5. '*' indicates a significant difference between *ABF3* or *NAC072* transgenic plants or mutants compared with WT plants under the same ABA treatment ($P<0.05$).

Table S1 The primers used in this study

Prime name	Prime sequence (5′ to 3′)
NAC072-F	5’-GGACATATGATGGGTGTTAGAGAGAAAGATCC-3’
NAC072-R	5’-GAAGGATCCTCATTGCCTAAACTCGAATGTTTGA-3’
NAC072ΔN-R	5’-GGAGGATCCTCACTGAGATCCAGATGTTTTCTTG-3’
NAC072ΔC-F	5’-GGACATATGATGAGACAAGCTGTTACTCCTGTTC-3’
P35S-F	5’-GGGGTACCCGGGCATGGAGATTCAAATAGAGGA-3’
P35S-R	5’-GCCTTGTCGACAGTCCCCCGTGTTCTCTC-3’
Pabf3-F	5’-GGGGTACCCGGGGGACTGTTCACCCATTCCCTT-3’
Pabf3-R	5’-GCCTTGTCGACTACTCAAGCTTTCGTACAACTAGG-3’
eGFP-F	5’- [illegible]
eGFP-R	5’-GGAAGATCTTATGGGTGTTAGAGAGAAAGATCC-3’
ABF3-F	5’- [illegible]
ABF3-R	5’-CCCTTGCTCACCATCCAGGGACCCGTCAATGTC-3’
mCherry-F	5’-CCTGGATGGTGAGCAAGGGCGAGGAG-3’
mCherry-R	5’-TTTGCTCCAGATCCTCTTCTGAGATGAGTTTTTGTTC-3’
6Myc-F	5’-AGGATCTGGAGCAAAAACTGATCTCAGAGGAAGATCTCGAGCAAAAGCTCATCTCAGAA-3’
6Myc-R	5’-TGCTCGAGGTCCTCCTCTGAGATCAGCTTTTGTTCCAGGTCTTCTTCTGAGATGAGCTT-3’
NAC072-RealT-F	5’-TTACGGTGGTTACGATGCG-3’
NAC072-RealT-R	5’-GAAACACCAAACCCACTCG-3’
ABF3-RealT-F	5’-CTTTGTTGATGGTGTGAGTGAG-3’
ABF3-RealT-R	5’-GTGTTTCCACTATTACCATTGC-3’
RD29A-RealT-F	5’-ATCACTTGGCTCCACTGTTGTTC-3’
RD29A-RealT-R	5’-ACAAAACACACATAAACATCCAAAGT-3’
RD29B-RealT-F	5’- AAAACGTTTGGAGGAAGATCC-3’
RD29B-RealT-R	5’- GGTACTCCAGCTTCTCCACCT-3’
Actin2-F	5’- GGTAACATTGTGCTCAGTGGTGG-3’
Actin2-R	5’- AACGACCTTAATCTTCATGCTGC-3’
Tublin- F	5’- ATCCGTGAAGAGTACCCAGAT-3’
Tublin- R	5’- AAGAACCATGCACTCATCAGC-3’
